# Investigating predictors of problematic alcohol, cannabis, and nicotine use among legal users of all three substances

**DOI:** 10.3389/fpsyt.2023.1110415

**Published:** 2023-02-23

**Authors:** Aaron Shephard, Şimal Dölek, Sean P. Barrett

**Affiliations:** Department of Psychology and Neuroscience, Dalhousie University, Halifax, NS, Canada

**Keywords:** cannabis, alcohol, nicotine, dependence, polysubstance use, SURPS

## Abstract

**Background:**

The three most used substances—alcohol, cannabis, and nicotine—are frequently concurrently. Use of each substance has been connected to an increased probability of use of the other substances, and the problematic use of each substance has been linked to demographic factors, substance use factors, and personality. However, little is known about which risk factors are most important for consumers of all three substances. This study examined the extent to which various factors are associated with dependence on alcohol, cannabis, and/or nicotine in users of all three substances.

**Methods:**

516 Canadian adults with past month use of alcohol, cannabis, and nicotine completed online surveys querying their demographics, personality, substance use history, and levels substance dependence. Hierarchical linear regressions were used to determine which factors best predicted levels of dependence on each substance.

**Results:**

Alcohol dependence was associated with levels of cannabis and nicotine dependence, and impulsivity, with 44.9% of variance explained. Cannabis dependence was predicted by alcohol and nicotine dependence levels, impulsivity, and the age of onset of cannabis use, with 47.6% of variance explained. Nicotine dependence was best predicted by alcohol and cannabis dependence levels, impulsivity, and dual use of cigarettes and e-cigarettes, with 19.9% of variance explained.

**Conclusions:**

Alcohol dependence, cannabis dependence, and impulsivity were the strongest predictors for dependence on each of the substances. A strong relationship between alcohol and cannabis dependence was evident, warranting further research.

## 1. Introduction

Problematic substance use is one of the most prevalent health issues in our society. In the last Canadian Alcohol and Drug Use Monitoring Survey (1), an estimated 6 million Canadians met the criteria for a substance use disorder. Statistics on polysubstance use are less accessible, but research has shown that dependent drug users report use of over three substances on average (2). Currently, the three most used substances in Canada are alcohol, cannabis, and nicotine (3, 4), all of which can be purchased legally. Although overall prevalence of use varies among substances [i.e., 76% of Canadians 15 years and older reported past year alcohol use (3), 27% of Canadians 16 years and older reported past year cannabis use (5), and 12 and 5% of Canadians 15 years and older reported past-30-day nicotine use *via* tobacco and vaping, respectively (4)], it is not uncommon for an individual to use two or more of these substances. Indeed, the use of each of these substances is related to increased probability of co-use of the other substances (6). Further, use of one substance may change the consumption amount and frequency of another: alcohol and cannabis co-use leads to increases in alcohol consumption (7, 8); smoking tobacco leads to increases in urges to smoke cannabis, as well as the inverse (9); smokers consume twice as much alcohol as non-smokers (10); and nicotine has been shown to increase alcohol consumption (11). As there is evidence that the concurrent use of nicotine, alcohol and cannabis may lead to increased use of each substance, gaining a better understanding of who is more likely to use and become dependent on these substances is imperative.

Problematic alcohol, cannabis, and nicotine use have been connected to a variety of risk factors in prior research. Age has been implicated as a factor for dependence in all three substances, with individuals often showing the most dependence symptoms in their late teens and early twenties (12). Age of onset has also been implicated in substance dependence, with earlier use of cigarettes being related to higher dependence scores (13). Other research has shown that, while a later age of onset was related to lower alcohol and cannabis use in general, individuals with a later age of onset co-used alcohol and cannabis more frequently than individuals with an earlier age of onset (14). While age of onset seems to relate to dependence on that substance, researchers have also investigated cross-substance age of onset; however, there appears to be little impact across substances, as neither age of onset of alcohol or nicotine use played a role in cannabis dependence, nor did age of onset of nicotine use play a role in alcohol dependence (13, 15).

Personality has also been implicated in dependence on alcohol, cannabis, and nicotine, particularly the traits measured by the Substance Use Risk Profile Scale (SURPS) (16). The SURPS assesses four personality traits: hopelessness/introversion, anxiety sensitivity, impulsivity, and sensation seeking. In one study, hopelessness, impulsivity, and sensation seeking positively correlated with drinking problems (17). Similarly, higher levels of hopelessness, impulsivity, and sensation seeking were related to polysubstance use (18). Contrarily, anxiety sensitivity appears to be inversely related with cannabis use (18) and drinking levels (17). Polysubstance users tend to be generally more impulsive than single substance users. Binge drinkers who met also used cannabis had higher impulsivity scores than those who did not use cannabis (19). Smokers who also reported additional substance use were more impulsive than individuals who only reported smoking (20, 21).

While there is evidence that all these factors play some role in substance dependence, the magnitude and importance of the role these factors play is unclear, as well as the commonality of the risk factors across different substance users. This is especially true when considering the use of all three substances by the same individual, as much of the literature focuses solely on use of a single substance. Similarly, much of the previous literature focuses on a single type of factor (e.g., demographics, personality, or age of onset). Combining these different types of factors into a larger model can both help to parse apart the roles of each type of factor on dependence, as well as the roles of each individual factor. The generalizability of past research is also brought into question because much of the research has been conducted on young and emerging adults, student, or in-patient populations, as opposed to the general population. It is for this reason that we chose to focus our research on a community sample of polysubstance (alcohol, cannabis, and nicotine) users across Canada. Thus, the purpose of this study is to determine the common risk factors for developing substance use within individuals who use alcohol, cannabis, and nicotine. While this research is mainly exploratory, we hypothesized that age of first use, impulsivity, and dependence on other substances will be key factors in predicting substance dependence.

## 2. Materials and methods

### 2.1. Participants and procedure

Canadian adults (19 years old or older; *N* = 516) were recruited, as part of a larger study, through Qualtrics Panels, a survey company with a large pool of potential participants. Researchers provided Qualtrics Panels with eligibility criteria, who then recruited participants to complete an online survey measuring demographic information, substance dependence, substance use history, and personality. Eligibility criteria included fluency in English and recreational use (past 30 days) of alcohol, cannabis, and nicotine (*via* cigarettes or e-cigarettes); potential participants who did not meet these criteria were screened out of the study. Participants were compensated for their part in the larger study. Mean participant age was 39.8 (*SD* = 12.4), there was roughly an equal number of males (*n* = 257) and females (*n* = 258), and most participants were Caucasian (69.8%). Regarding education, 26.2% had completed community or technical college and 41.7% had obtained a university degree.

### 2.2. Materials

Alcohol dependence was measured *via* the Alcohol Use Disorders Identification Test (AUDIT) (22), a 10-item self-report measure that identifies frequency and quantity of alcohol use, dependence symptoms, and alcohol-related consequences. The AUDIT has a high test-retest reliability and satisfactory internal consistency (23). Consistent with the literature, scores of 15 and above were considered indicative of alcohol dependence (22), with 161 (31.2%) of participants meeting this criteria.

Cannabis dependence was measured *via* the Cannabis Use Disorder Identification Test- Revised (CUDIT-R) (24), an 8-item self-report measure that assesses problematic cannabis use within the past 6 months. The CUDIT-R has been used in a variety of cannabis subpopulations and has been found to be both valid and reliable (25, 26). Consistent with the literature, scores of 13 and above were considered indicative of cannabis dependence (24), with 150 (29.1%) of participants meeting this criteria.

Nicotine dependence was assessed using both the Fagerström Test for Cigarette Dependence (FTCD) (27) and the e-cigarette Fagerström Test of Cigarette Dependence [e-FTCD; (28)], depending on whether the participant use cigarettes, e-cigarettes, or both. Participants who only used e-cigarettes were directed to complete E-cigarette Fagerström Test of Cigarette Dependence (e-FTCD) (28). The FTCD is a 6-item self-report measure that assesses a person's level of cigarette dependence. The FTCD is commonly used in nicotine and tobacco research and has good reliability and validity (27). The e-FTCD is a 6-item adapted version of the FTCD, modified by changing all references of cigarettes to e-cigarettes and all references of smoking to vaping. The e-FTCD has been proven reliable and valid for use with e-cigarette users (28). Scores of 4 and above were considered indicative of moderate nicotine dependence (29), with 292 (56.6%) participants meeting this criteria.

Personality traits of the participants were measured *via* the Substance Use Risk Profile Scale (SURPS) (16), a 23-item questionnaire that measures 4 distinct personality risk factors based on reinforcement-sensitivity models of substance use: hopelessness/ introversion (HI); anxiety sensitivity (AS); impulsivity (IMP), and sensation seeking (SS). The SURPS has been shown to have adequate psychometric properties (16).

### 2.3. Statistical analyses

All statistical analyses were conducted using R version 4.2.1; there were no missing data in the analyses. To compare participants regardless of their preferred nicotine consumption method (cigarettes or e-cigarettes), a composite nicotine dependence variable was created by taking the highest value of the FTCD and e-FTCD scores; this gave each participant a single nicotine dependence score. Age of onset of regular use for each of the substances was calculated by subtracting reported years of regular use from current age; a composite nicotine age of onset was created by taking the lowest age between cigarette and e-cigarette age of onset variables. Three different hierarchical multiple regressions were conducted, in which dependent variables were levels of dependence on alcohol, cannabis, and nicotine, respectively. In the regression analyses, predictor variables were added in four stages: the first models contained demographic variables (age, sex, and education), the second models contained personality variables (hopelessness, anxiety sensitivity, impulsivity, and sensation seeking), the third models contained ages of onset of regular use across substances, and the fourth models contained dependence on the other two substances. Regarding nicotine dependence, the fourth model included an additional variable: dual use of cigarettes and e-cigarettes. The variables were added in this order as it was the most chronologically plausible order, as demographic variables are from birth, personality is mostly stable from childhood, and age of first use comes before substance dependence. Finally, *post-hoc* analyses were conducted on significant variables in the final models for each of the regressions. Participants were categorized by dependence on each substance (dependent vs. non-dependent) using the cut-offs described in Section 2.2. For continuous variables, the means of each group were compared *via* Welch's *t*-tests; for categorical variables (e.g., sex and dual use of cigarettes/e-cigarettes), the ratios were compared *via* Pearson's χ^2^ tests.

## 3. Results

### 3.1. Alcohol dependence

**Figure 1:**
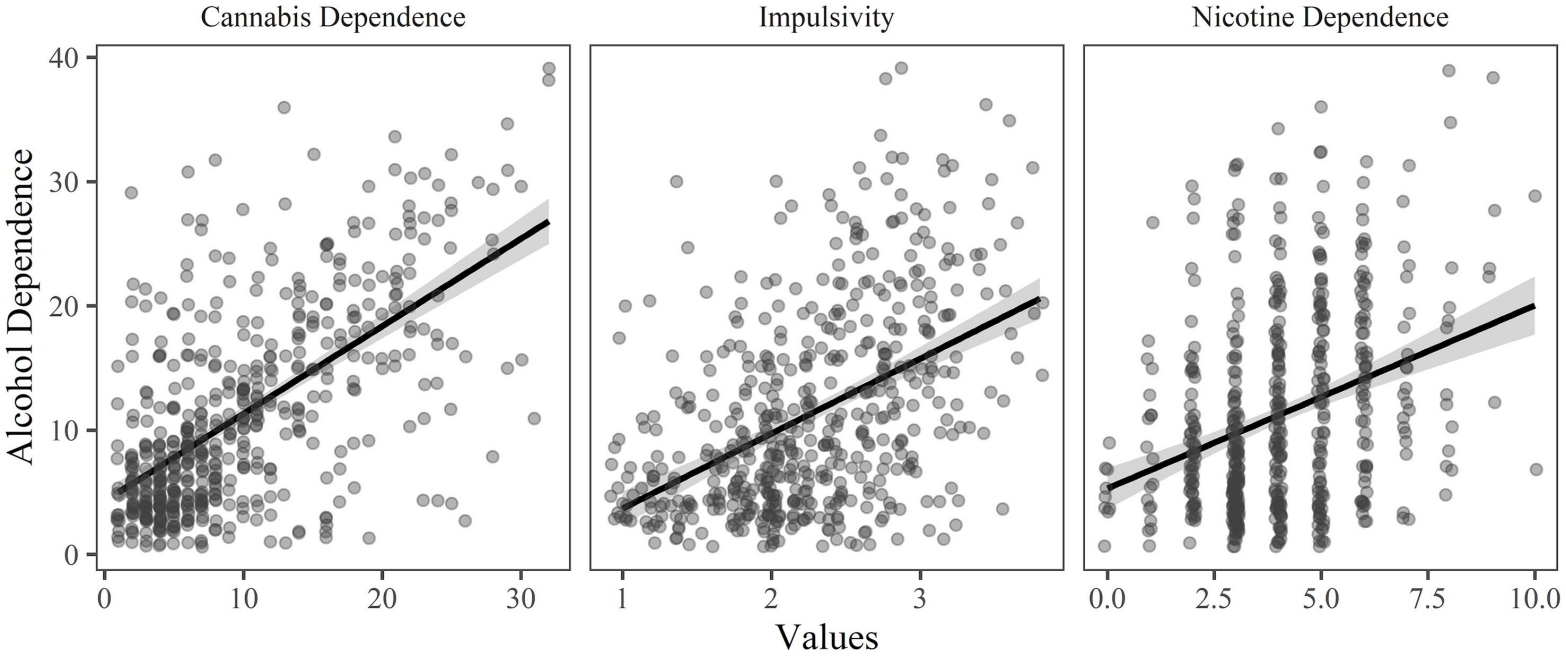
The relationship between alcohol dependence and each of the significant predictor variables (*p* < 0.05 and relative importance ≥ 0.02) in the final model of the hierarchical regression, holding all other variables constant.

A four-step hierarchical multiple regression was conducted with level of alcohol dependence as the dependent variable (see Table 1). The analyses revealed that demographics contributed significantly to the regression model, *F*_(3, 511)_ = 19.009, *p* < 0.001, and accounted for 6.3% of the variance in alcohol dependence. Adding personality explained a further 19.4% of the variance, *F*_(4, 507)_ = 44.169, *p* < 0.001. Adding age of onset for each substance did not significantly improve the model, *F*_(3, 504)_ = 0.658, *p* = 0.579, but adding dependence on other substances explained a further 19.0% of the variance in alcohol dependence, *F*_(2, 502)_ = 86.540, *p* < 0.001. In the final model, the significant predictor variables were cannabis dependence (explaining 23.7% of the variance), impulsivity (explaining 9.0% of the variance), nicotine dependence (explaining 3.9% of the variance), cannabis age of onset (explaining 1.0% of the variance), and sex (explaining 0.9% of the variance). See Figure 1 for the relationship between the significant predictor variables (excluding sex) and alcohol dependence.

**Table 1:** Hierarchical multiple regression statistics for alcohol dependence.

	**Model 1— Demographics**			**Model 2— Personality**			**Model 3— Age of onset**			**Model 4— Substances**			
**Predictors**	***B***	***SE***	***p***	***B***	***SE***	***p***	***B***	***SE***	***p***	***B***	***SE***	***p***	***RI***
Age	−0.14	0.03	**< 0.001**	−0.06	0.03	**0.017**	−0.05	0.03	0.098	−0.05	0.03	0.071	0.012
Sex	2.35	0.70	**0.001**	2.04	0.64	**0.001**	1.99	0.64	**0.002**	1.14	0.56	**0.042**	0.009
Education	−0.00	0.31	0.993	−0.20	0.28	0.466	−0.17	0.29	0.544	−0.11	0.25	0.645	< 0.001
Hopelessness				0.92	0.56	0.104	0.90	0.57	0.112	0.40	0.49	0.418	0.006
Anxiety sensitivity				0.00	0.56	0.994	0.02	0.56	0.968	−0.21	0.49	0.671	0.004
Impulsivity				4.85	0.63	**< 0.001**	4.91	0.63	**< 0.001**	2.34	0.58	**< 0.001**	**0.090**
Sensation seeking				1.56	0.66	**0.019**	1.44	0.67	**0.033**	1.01	0.58	0.084	0.038
Alcohol age of onset							−0.04	0.04	0.348	−0.07	0.04	0.071	0.004
Cannabis age of onset							−0.01	0.03	0.701	0.08	0.03	**0.007**	0.010
Nicotine age of onset							0.01	0.04	0.870	−0.01	0.03	0.796	< 0.001
Cannabis dependence										0.56	0.05	**< 0.001**	**0.237**
Nicotine dependence										0.43	0.17	**0.010**	**0.039**
Observations	515			515			515			515			
*R*^2^/Δ*R*^2^	0.063			0.257 /0.194			0.259 /0.002			0.449 /0.190			

RI, relative importance. Bold p values indicate values of p < 0.05; bold RI values indicate values over 0.02.

### 3.2. Cannabis dependence

**Figure 2:**
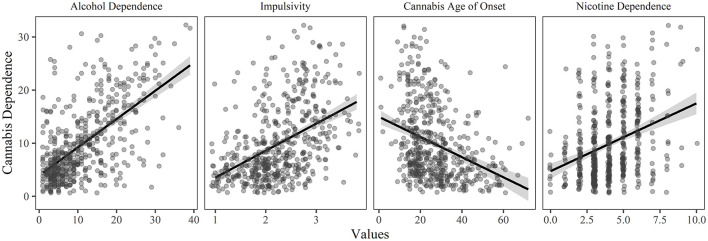
The relationship between cannabis dependence and each of the significant predictor variables (*p* < 0.05 and relative importance ≥ 0.02) in the final model of the hierarchical regression, holding all other variables constant.

A four-step hierarchical multiple regression was conducted with level of cannabis dependence as the dependent variable (see Table 2). The analyses revealed that demographics contributed significantly to the regression model, *F*_(3, 511)_ = 21.557, *p* < 0.001, and accounted for 6.8% of the variance in cannabis dependence. Adding personality explained a further 17.8%, *F*_(4, 507)_ = 42.635, *p* < 0.001, adding age of onset for each substance explained an additional 4.8%, *F*_(3, 504)_ = 15.261, *p* < 0.001, while adding dependence on other substances explained a further 18.2% of the variance *F*_(2, 502)_ = 87.089, *p* < 0.001. In the final model, the significant predictor variables were alcohol dependence (explaining 23.1% of the variance), impulsivity (explaining 7.9% of the variance), cannabis age of onset (explaining 6.1% of the variance), nicotine dependence (explaining 3.8% of the variance), and alcohol age of onset (explaining 0.4% of the variance). See Figure 2 for the relationship between the significant predictor variables and cannabis dependence.

**Table 2:** Hierarchical multiple regression statistics for cannabis dependence.

	**Model 1— Demographics**			**Model 2— Personality**			**Model 3— Age of onset**			**Model 4— Substances**			
**Predictors**	***B***	***SE***	***p***	***B***	***SE***	***p***	***B***	***SE***	***p***	***B***	***SE***	***p***	***RI***
Age	−0.14	0.02	**< 0.001**	−0.07	0.02	**0.001**	−0.01	0.03	0.827	0.001	0.02	0.519	0.014
Sex	1.57	0.60	**0.010**	1.37	0.56	**0.015**	1.31	0.54	**0.017**	0.39	0.48	0.407	0.004
Education	−0.11	0.27	0.677	−0.25	0.24	0.304	−0.07	0.24	0.773	0.02	0.21	0.930	< 0.001
Hopelessness				1.01	0.49	**0.041**	0.85	0.48	0.079	0.45	0.42	0.277	0.008
Anxiety sensitivity				0.11	0.49	0.817	0.16	0.48	0.740	0.02	0.41	0.956	0.005
Impulsivity				4.01	0.55	**< 0.001**	4.03	0.54	**< 0.001**	1.78	0.49	**< 0.001**	**0.079**
Sensation seeking				1.17	0.58	**0.043**	0.69	0.57	0.225	0.08	0.50	0.876	0.028
Alcohol age of onset							0.05	0.04	0.180	0.06	0.03	**0.037**	0.004
Cannabis age of onset							−0.17	0.03	< 0.0**01**	−0.16	0.02	**< 0.001**	**0.061**
Nicotine age of onset							0.05	0.03	0.171	0.05	0.03	0.072	0.003
Alcohol dependence										0.40	0.03	**< 0.001**	**0.231**
Nicotine dependence										0.39	0.14	**0.006**	**0.038**
Observations	515			515			515			515			
*R*^2^/Δ*R*^2^	0.068			0.246 /0.178			0.294 /0.048			0.476 /0.182			

RI, relative importance. Bold p values indicate values of p < 0.05; bold RI values indicate values over 0.02.

### 3.3. Nicotine dependence

**Figure 3:**
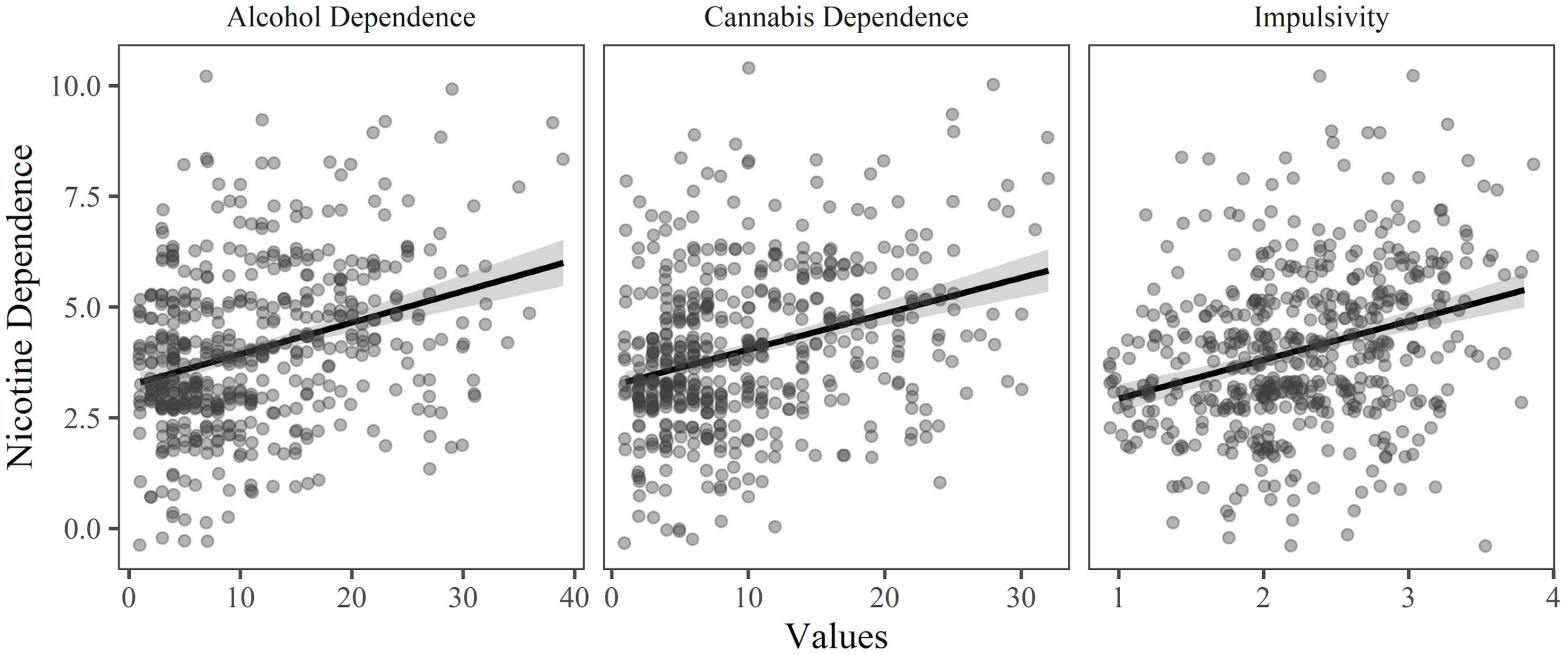
The relationship between nicotine dependence and each of the significant predictor variables (*p* < 0.05 and relative importance ≥ 0.02) in the final model of the hierarchical regression, holding all other variables constant.

A four-step hierarchical multiple regression was conducted with nicotine dependence as the dependent variable (see Table 3). The analyses revealed that demographics contributed significantly to the regression model, *F*_(3, 511)_ = 3.362, *p* = 0.019, and accounted for 1.6% of the variance, adding personality explained a further 10.0%, *F*_(4, 507)_ = 15.653, *p* < 0.001, adding age of onset for each substance explained an additional 1.6% *F*_(3, 504)_ = 3.230, *p* = 0.022 while, adding dependence on other substances and dual use of cigarettes/e-cigarettes explained an additional 6.7% of the variance in nicotine dependence, *F*_(3, 501)_ = 14.031, *p* < 0.001. In the final model, the significant predictor variables were alcohol dependence (explaining 4.0% of the variance), cannabis dependence (explaining 4.0% of the variance), impulsivity (explaining 3.6% of the variance), dual use of cigarettes and e-cigarettes (explaining 2.8% of the variance), anxiety sensitivity (explaining 1.8% of the variance), and nicotine age of onset (explaining 1.4% of the variance). See Figure 3 for the relationship between the significant predictor variables (excluding dual use) and nicotine dependence.

**Table 3:** Hierarchical multiple regression statistics for nicotine dependence.

	**Model 1— Demographics**			**Model 2— Personality**			**Model 3— Age of onset**			**Model 4—Substances**			
**Predictors**	***B***	***SE***	***p***	***B***	***SE***	***p***	***B***	***SE***	***p***	***B***	***SE***	***p***	***RI***
Age	−0.02	0.01	**0.018**	−0.00	0.01	0.562	−0.00	0.01	0.925	0.01	0.01	0.342	0.002
Sex	0.28	0.16	0.079	0.30	0.15	**0.048**	0.28	0.15	0.064	0.17	0.15	0.240	0.003
Education	−0.06	0.07	0.374	−0.09	0.07	0.181	−0.05	0.07	0.484	−0.08	0.07	0.232	0.002
Hopelessness				0.11	0.13	0.400	0.07	0.13	0.588	0.05	0.13	0.711	0.003
Anxiety sensitivity				0.31	0.13	**0.020**	0.33	0.13	**0.014**	0.36	0.13	**0.005**	**0.018**
Impulsivity				0.70	0.15	**< 0.001**	0.71	0.15	**< 0.001**	0.39	0.16	**0.013**	**0.036**
Sensation seeking				0.12	0.16	0.444	0.09	0.16	0.552	−0.00	0.15	0.999	0.009
Alcohol age of onset							−0.00	0.01	0.890	−0.00	0.01	0.704	0.001
Cannabis age of onset							−0.00	0.01	0.674	0.00	0.01	0.706	0.003
Nicotine age of onset							−0.02	0.01	**0.011**	−0.02	0.01	**0.015**	**0.014**
Cannabis dependence										0.03	0.01	**0.015**	**0.040**
Alcohol dependence										0.03	0.01	**0.019**	**0.040**
Dual user										0.56	0.15	**< 0.001**	**0.028**
Observations	515			515			515			515			
*R*^2^/Δ*R*^2^	0.016			0.116/0.100			0.132/0.016			0.199/0.067			

RI, relative importance. Bold p values indicate values of p < 0.05; bold RI values indicate values over 0.02.

### 3.4. *Post-hoc* analyses

*Post-hoc* analyses were conducted for each model, comparing the means of the significant variables in each final model, with participants being categorized by level of dependence (dependent vs. non-dependent). For the alcohol dependence model, *post-hoc* analyses showed significant differences between the dependent and non-dependent groups for each of the significant predictor variables in the final model (all *p*'s < 0.03). For the cannabis dependence model, *post-hoc* analyses showed significant differences (*p*'s < 0.001) between the dependent and non-dependent groups for each of the significant predictor variables in the final model except alcohol age of onset (*p* = 0.401). Finally, for the nicotine dependence model, *post-hoc* analyses showed significant differences between the dependent and non-dependent groups for each of the significant predictor variables in the final model (all *p*'s ≤ 0.002). See Table 4 for detailed results of the *post-hoc* analyses.

**Table 4:** *Post-hoc t-tests* comparing significant predictors between dependent and non-dependent users.

	**Dep**.	**Non-Dep**.						**95% CI**	
**Variables**	***m (**σ**)***	***m (**σ**)***	***t***	***df***	***p***	***MD***	***SE***	**Lower**	**Upper**
**Alcohol dependence (a score of 15 or higher on the AUDIT)**									
	*n* = 161	*n* = 355							
Cannabis dependence	15.49 (7.38)	7.32 (5.13)	12.74	232.91	< 0.001	8.17	0.64	6.91	9.44
Impulsivity	2.62 (0.57)	2.08 (0.58)	9.91	309.94	< 0.001	0.54	0.05	0.43	0.65
Nicotine dependence	4.74 (1.78)	3.70 (1.68)	6.226	293.14	< 0.001	1.04	0.17	0.71	1.37
Cannabis age of onset	24.41 (10.47)	27.90 (13.05)	−3.24	379.68	0.001	−3.49	1.08	−5.60	−1.37
**Cannabis dependence (a score of 13 or higher on the CUDIT-R)**									
	*n* = 150	*n* = 366							
Alcohol dependence	18.11 (8.47)	8.41 (5.95)	12.79	211.97	< 0.001	9.70	0.76	8.20	11.19
Impulsivity	2.66 (0.54)	2.08 (0.58)	10.88	297.70	< 0.001	0.58	0.05	0.48	0.69
Cannabis age of onset	21.95 (10.21)	28.80 (12.68)	−6.43	341.18	< 0.001	−6.84	1.07	−8.94	−4.75
Nicotine dependence	4.83 (1.72)	3.69 (1.69)	6.82	272.76	< 0.001	1.13	0.17	0.81	1.46
Alcohol age of onset	20.83 (7.84)	21.50 (8.81)	−0.84	309.45	0.401	−0.66	0.79	−2.22	0.89
**Nicotine dependence (a score of 4 or higher on the FTCD/eFTCD)**									
	*n* = 292	*n* = 224							
Alcohol dependence	13.18 (8.45)	8.68 (6.79)	6.70	512.85	< 0.001	4.50	0.67	3.18	5.82
Cannabis dependence	11.51 (7.37)	7.72 (5.91)	6.47	512.96	< 0.001	3.79	0.59	2.64	4.94
Impulsivity	2.39 (0.63)	2.07 (0.58)	5.95	494.80	< 0.001	0.32	0.05	0.21	0.42
Anxiety sensitivity	2.79 (0.58)	2.62 (0.59)	3.18	473.63	0.002	0.17	0.05	0.06	0.27
Nicotine age of onset	20.54 (8.86)	23.00 (8.68)	−3.16	484.76	0.002	−2.46	0.78	−3.99	−0.93

As sex and dual use are dichotomous variables, Pearson's χ^2^ analyses were conducted to compare difference in these variables as opposed to Welch's t-tests. The test was significant for sex in the alcohol dependence model, $\chi_{\left(1\right)}^{2}$ = 5.05, p = 0.025, with alcohol dependent individuals more likely to be male when compared to non-dependent individuals [OR = 1.54, 95% CI (1.06, 2.24)]. The test was also significant for dual use in the nicotine dependence model, $\chi_{\left(1\right)}^{2}$ = 19.92, p < 0.001, with nicotine dependent individuals more likely to be dual users of cigarettes and e-cigarettes than single users of either [OR = 1.88, 95% CI (1.32, 2.67)].

## 4. Discussion

This study aimed to determine risk factors for developing substance dependence among individuals who use alcohol, cannabis, and nicotine. After including a variety of predictor variables, including demographic variables, personality, age of onset for the three substances, and dependence on the other substances, our analyses revealed several risk factors for dependence on each of the substances. Common predictor variables were responsible for large amounts of variance in each model; specifically, impulsivity and dependence on the other substances were key risk factors for elevated levels of dependence on each of alcohol, cannabis, and nicotine. The role of impulsivity in these models confirms prior research in the field as numerous studies highlight the connection between impulsivity and substance dependence (30–34).

Particularly of note was the strong relationship between levels of cannabis and alcohol dependence. In the model for alcohol dependence, level of cannabis dependence alone predicted 23.7% of the variance; while in the model for cannabis dependence, level of alcohol dependence predicted 23.1% of the variance. When using Cohen's (35) definition for effect sizes, these are both substantial effect sizes; as such, it appears that there is a considerable relationship between levels of cannabis and alcohol dependence in our sample. These findings are consistent with evidence of a positive relationship between cannabis dependence with alcohol problems (36, 37), but to our knowledge are the first to demonstrate an association in a community sample of recreational users in a jurisdiction where both substances are legally available. More research should be done to further investigate the link between cannabis and alcohol use and dependence, and to determine the extent to which public health policy contributes to this association.

While we were able to explain 44.9 and 47.6% of the variance in alcohol and cannabis dependence, we were only able to explain 19.9% of the variance in nicotine dependence. This was noteworthy, as prior research has shown a strong connection between nicotine and alcohol use (10, 38, 39), nicotine and cannabis use (40, 41), and nicotine and anxiety sensitivity (42–44). For this study, we included participants who used cigarettes, e-cigarettes, or both cigarettes and e-cigarettes. It is possible that including only one method of nicotine consumption may have led to different results for nicotine dependence, as studies have shown differing levels of dependence between cigarette and e-cigarette users (45–47). However, follow-up analyses were conducted to explore the difference in variance explained in those who used cigarettes vs. those who used e-cigarettes; the variance explained by each model remained fairly stable (within 2% of variance explained). Thus, there must be other key variables in nicotine dependence that we failed to account for in our analyses.

This study is not without limitations, particularly with respect to methodological considerations. First, participants were recruited *via* Qualtrics panels, an online survey platform. While online panel data has been shown to be an adequate source for exploratory research, questions remain about the generalizability of research conducted *via* these platforms, particularly with respect to data quality. However, a recent meta-analysis found no significant differences in effect sizes between data collected *via* online panels and more traditional methods (48). Additionally, several data quality checks (e.g., speeder checks and screening) were in place to help collect the best quality data possible. Second, to meet eligibility criteria, participants were required to use all three substances; as such, we may have decreased the possibility of explaining connections between only two of the substances (e.g., the relationship between nicotine and alcohol). Similarly, while participants were required to use all three substances, we did not ask about or measure other substance use, nor did we measure what their overall substance of choice was. Inclusion of other substances may have led to a more fulsome explanation of the variance in substance dependence levels, whereas substance of choice may have had potential implications for level of dependence on each substance. Additionally, while we included participants who used each substance as little as once per month to as much as daily, it is important to note that these findings may not generalize to groups who use on a less frequent basis. Also, as the study was conducted in a jurisdiction where all three substances are legal, findings may not be generalizable to jurisdictions with different levels of legalization. Finally, as with all cross-sectional studies, the relationship between variables does not infer causation; the direction of effects between the predictors and outcomes in our models is not measured in this study, only the strength of the relationship.

## 5. Conclusion

In conclusion, this study demonstrates a strong relationship between alcohol dependence, cannabis dependence, impulsivity, and, to a lesser extent, nicotine dependence in Canadians who use all three substances. Findings suggest that common risk factors may increase vulnerability for substance misuse in general and for dependence on alcohol and cannabis, in particular.

## Data availability statement

The raw data supporting the conclusions of this article will be made available by the authors, without undue reservation.

## Ethics statement

The studies involving human participants were reviewed and approved by Dalhousie University Health Sciences Research Ethics Board. The patients/participants provided their written informed consent to participate in this study.

## Author contributions

AS: conceptualization, methodology, project administration, statistical analyses, writing—original draft, review and editing, and supervision. ŞD: statistical analyses and writing—original draft. SB: conceptualization, methodology, project administration, writing—review and editing, supervision, and funding acquisition. All authors approved the final version of this manuscript.
